# Multifocal leukoencephalopathy associated with intensive use of cocaine and the adulterant levamisole in a 29-year old patient

**DOI:** 10.1186/s42466-022-00202-y

**Published:** 2022-08-01

**Authors:** Nadine Tollens, Philip Post, Michael Martins Dos Santos, Pascal Niggemann, Melanie Warken, Joachim Wolf

**Affiliations:** 1 Diakonissenkrankenhaus Mannheim, Steubenstraße 91-93, 68199 Mannheim, Germany; 2 Zentralinstitut für seelische Gesundheit Mannheim, J5, 68159 Mannheim, Germany; 3 Radiologie Nuklearmedizin Mannheim, Steubenstraße 91-93, 68199 Mannheim, Germany

**Keywords:** Levamisole, Leukoencephalopathy, White matter lesions, Neurological symptoms, Cocaine use, Adulterant, Antihelminthic drug

## Abstract

Levamisole is a common adulterant of cocaine and has been associated with reversible leukoencephalopathy in cocaine users. We report a case of two episodes with severe neurological symptoms and multifocal white matter lesions with brainstem and cerebellar involvement in a 29-year-old man after sporadic cocaine consumption. A urinalysis was positive for levamisole. Neurological deficits as well as MRI presentation improved after cessation of levamisole exposure and two courses of intravenous high-dose glucocorticoid therapy. Early diagnosis of levamisole-induced multifocal leukoencephalopathy and treatment with corticosteroids without delay is essential for a good recovery from neurological symptoms. Although cocaine is one of the most prevalent abused illicit drugs, cocaine- and levamisole-induced multifocal leukoencephalopathy is underdiagnosed as this disorder is not often described in the literature and anamnesis of drug abuse is not admitted by the patient. Therefore, an additional screening for cocaine and levamisole in clinical practice is useful in similar cases to support the diagnosis.

## Background

There is broad consensus about different cocaine-induced brain injuries including ischemic and hemorrhagic strokes and vasculitis as a consequence of vascular pathology, as well as acute and subacute cocaine-induced leukoencephalopathy resulting from metabolic changes [1, 2]. Additionally, another type of cocaine-induced encephalopathy due to a postulated immune-mediated mechanism was described in a small number of cases associated with cocaine abuse. An adulterant, levamisole, is commonly found in samples of seized cocaine. According to statements from the State Office of Criminal Investigation in Baden-Wuerttemberg^1^ (Germany), levamisole has been used as an adulterant for about 15 years and was proven by a nonquantitative measurement in around every third investigated sample in 2020. Initially introduced as an antihelminthic drug to treat parasitic worm infections in humans and livestock, therapeutic use of levamisole in humans has been discontinued due to severe adverse reactions, including agranulocytosis, tissue necrosis and leukoencephalopathy [3–5]. Apart from merely increasing the volume of cocaine in a manner undetectable for the consumer, the metabolic pathways for levamisole in humans lead to aminorex, a stimulant in its own right, which might lead to prolonged psychotropic effects of adulterated cocaine and amphetamine-like psychostimulatory properties increasing monoamine release [2]. The number of cases of patients who suffer a levamisole-induced leukoencephalopathy is likely underestimated [6–9].

We report the case of a 29-year-old cocaine user from Germany who presented in an emergency room with reduced vigilance. MRI showed multifocal white matter lesions, urinalysis yielded proof of levamisole. After an initial course of intravenous steroids, the patient regained consciousness while showing a persisting mild cognitive impairment. A successive deterioration of clinical presentation necessitated a second course of intravenous glucocorticoids.

## Case presentation

**Fig. 1:**
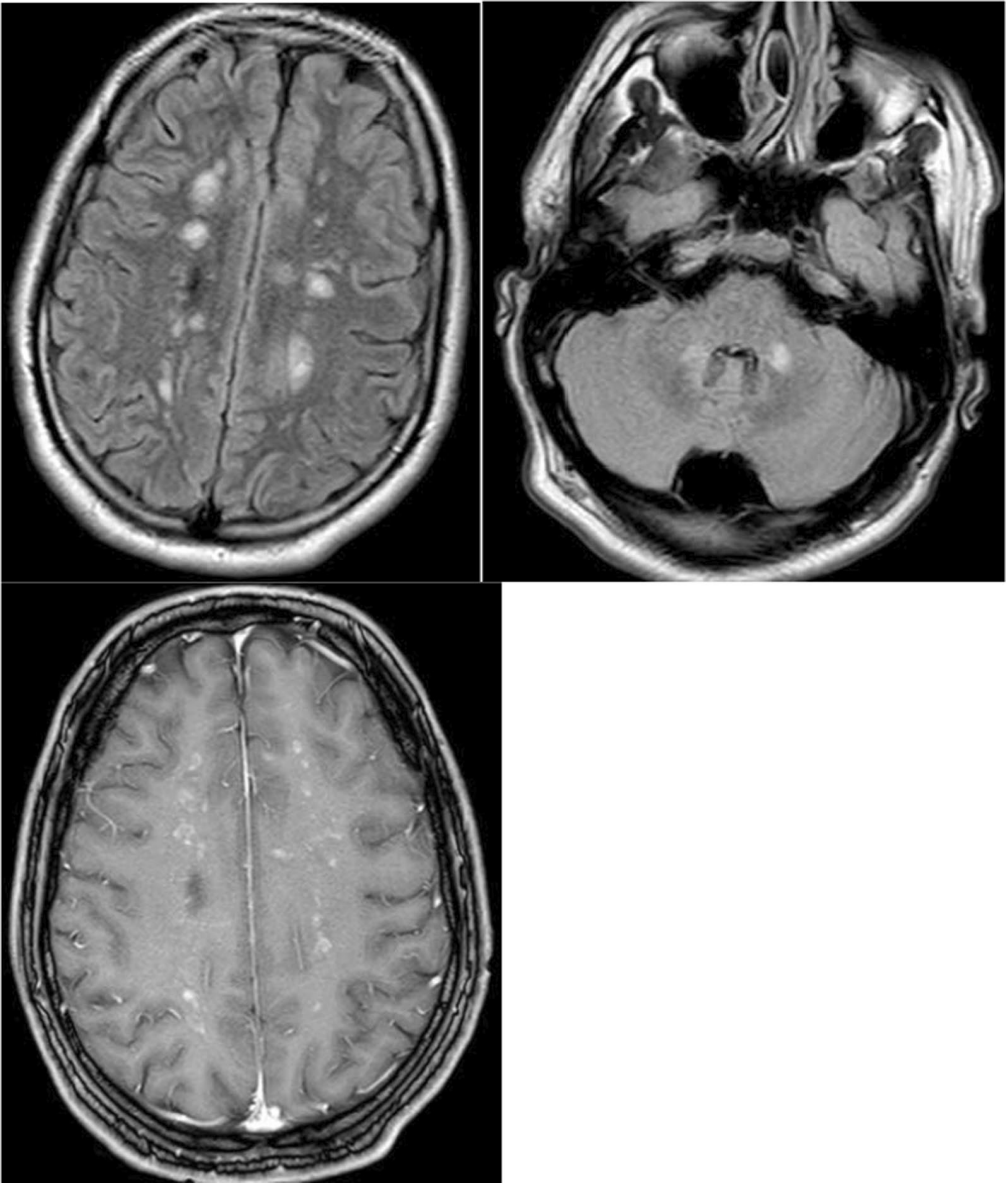
MRI at admission. The FLAIR sequence depicts multiple hyperintense infra- and supratentorial white matter lesions. Patchy contrast enhancement of all lesions is visible on contrast enhanced T1 weighted imaging

A 29-year-old man was presented to the emergency room by his family with insufficient food consumption, apathy and personality change for 2 days. No hints for trauma, vaccination or drug use were reported. The medical examination revealed a restless patient with non-fluent aphasia and impaired speech comprehension. An MRI study of the brain demonstrated infra- and supratentorial white matter lesions with patchy contrast enhancement of all lesions on the contrast enhanced T1 weighted imaging (Fig. 1). Initial urinalysis was positive for cocaine and benzodiazepines, further quantitative analysis beyond the general drug screening confirmed the presence of levamisole. Another detailed anamnesis revealed an intensive transnasal consumption of cocaine over the preceding 2 months. Diagnostics concerning infectious, autoimmune and metabolic differential diagnoses were normal or negative with respect to the following parameters: complete blood count, serum electrolytes, urea, creatinine, glucose, antinuclear antibodies, perinuclear and cytoplasmic anti-neutrophil cytoplasmic antibodies (pANCA and cANCA respectively), antibodies against extractable nuclear antigens, thyroid stimulating hormone level, free T3 and T4, viral serology for HIV/CMV/EBV/Measles/Mumps/Rubella, Lyme serology, syphilis screen. Liver enzymes and C-reactive protein were mild to moderately elevated. Cerebrospinal fluid (CSF) results included a normal protein and glucose concentration, normal total cell count. The oligoclonal bands were positive with proof of two bands in the isoelectric focusing and a positive MRZ-reaction was detected.

After a treatment with intravenous methylprednisolone (1000 mg daily for five consecutive days) the patient’s neurological condition improved, while showing persistent mild cognitive impairment. Monitoring of vital parameters showed intermittent periods of self-limiting bradycardia. Ten days after the admission, the patient left the hospital, against clinical advice and refused a further treatment in a neurorehabilitation hospital.

**Fig. 2:**
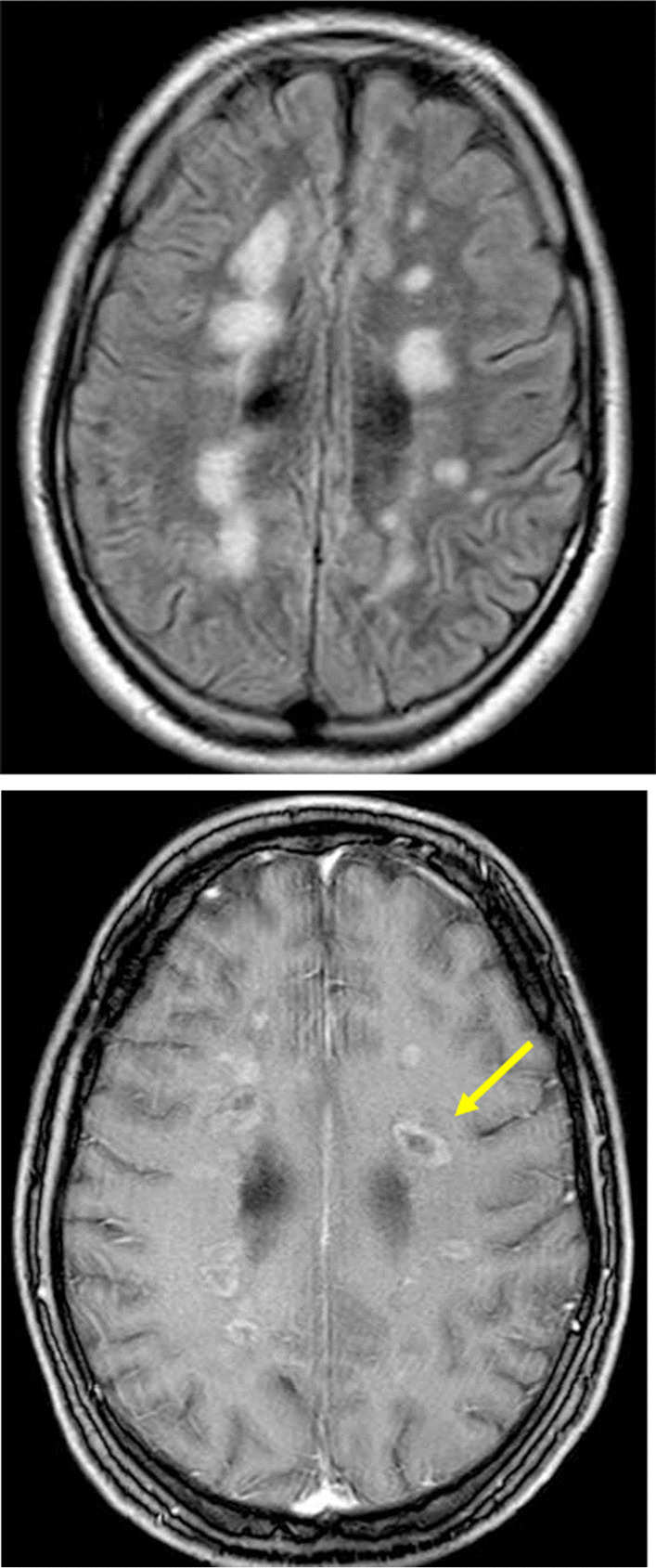
Follow-up MRI 2 weeks after initial admission shows an increase in size of the white matter lesions on the FLAIR sequence, representing further swelling of the brain tissue. Most lesions showed a ring shaped contrast enhancement (Arrow)

Two weeks later, the patient presented again because of apathy, dizziness, reduced intake of nutrition and markedly impaired cognitive function with disorganized behaviour. A renewed consumption of cocaine ad interim was denied trustworthily. cMRI with angiography showed an augmentation of white matter lesions (Fig. 2). Another CSF was inconspicuous in terms of cell count and protein concentration. Oligoclonal bands were again positive while additional results (soluble IL-2 receptor, aquaporin-4-AAC, toxoplasmosis serology) were normal.

A second course of intravenous glucocorticoid therapy with 2000 mg methylprednisolone per day for 3 days followed by 1000 mg per day for 3 days was initiated. This approach represented a therapeutic attempt based on the observed improvement of symptoms in the first episode and the recommended therapy in the event of a relapse of multiple sclerosis with persistent deficits, since no established therapy schemes are described in the literature for levamisole-induced leukoencephalopathy. Afterwards symptoms improved clearly. However, the patient continued to present cognitive deficits and a disorder for formal thoughts with deceleration of answers and reactions, a prolonged response latency, deficits in comprehension and reduced ability to concentrate as well as intermittent hypomanic symptoms in the form of excessive generosity, increased pressure of speech and hyperactivity.

**Fig. 3:**
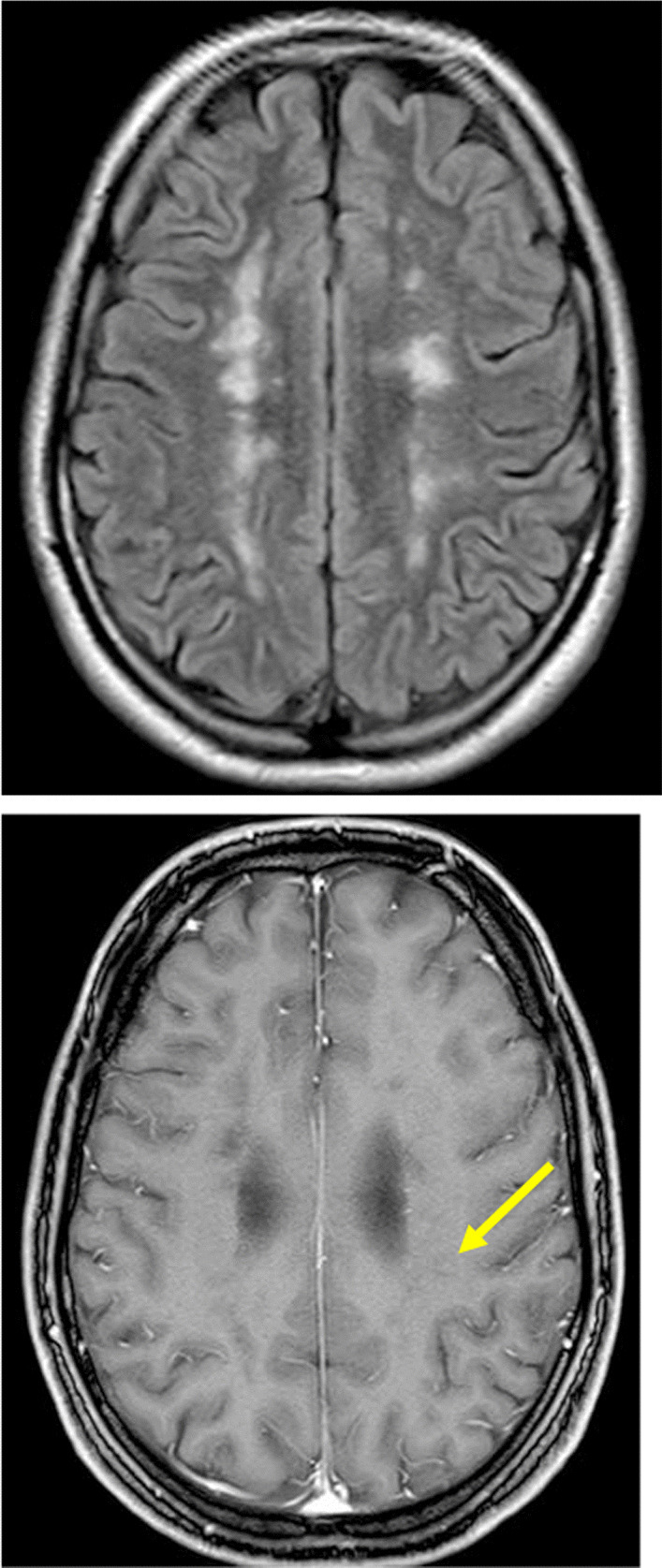
Follow-up 4 weeks after initial presentation: The FLAIR sequence depicts a reduction in size of the white matter lesions. Contrast enhancement is markedly reduced with residual patchy enhancement in the area of multifocal leukoencephalopathy (Arrow)

**Fig. 4:**
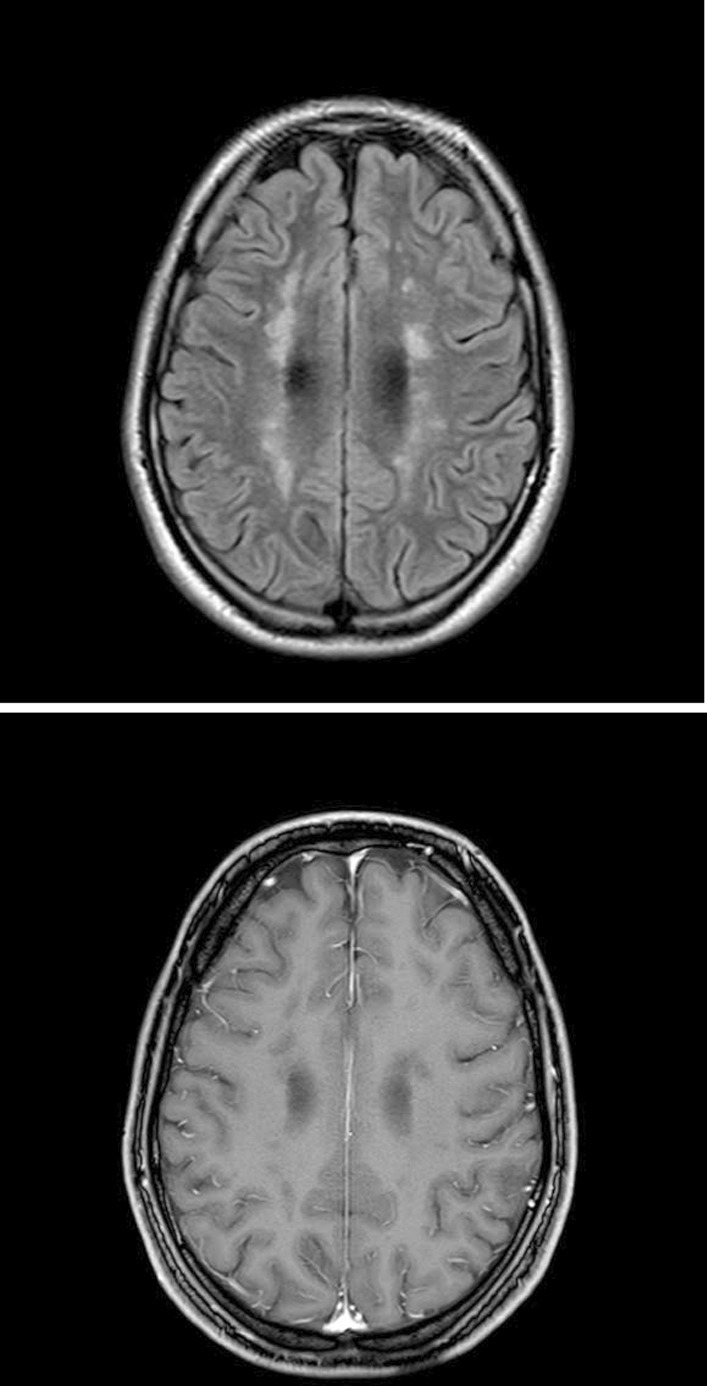
Follow-up 3 months after initial presentation: The FLAIR sequence depicts a reduction in size of the white matter lesions without pathological/corresponding contrast enhancement

The patient was discharged to inpatient rehabilitation after a 2-week inpatient stay. Four weeks later, an outpatient cMRI follow-up was carried out, which showed a clear improvement with a decreased size of the white matter lesions. Contrast enhancement was still present albeit with lesser degree of enhancement (Fig. 3). A repeat cMRI 3 months after the first symptoms demonstrated complete remission of the contrast enhancement (Fig. 4). Additionally, expanded cerebrospinal fluid space was observed in the baseline MRI indicating beginning brain atrophy, which did not increase over the follow-up of 2 months.

At the outpatient presentation in domo for clinical follow-up 3 months after discharge, the condition has improved significantly with a regain of 99% of its previous state according to the patient's subjective assessment. Nocturnal sleep disorder with increased daytime sleepiness and a fluctuating forgetfulness were reported by the patient. In the clinical examination neurological deficits were not found and significant cognitive deficits were not detectable.

## Diagnosis, discussion and conclusions

Based on the imaging findings and the patient's anamnesis, we diagnosed multifocal inflammatory leukoencephalopathy after excessive cocaine abuse, most likely caused by the cocaine alternant levamisole or a direct toxic effect by the drug itself. Levamisole causes an inflammatory response due to the migration of monocytes and macrophages so that an autoinflammatory genesis of the toxic effect must be considered [10]. Active demyelination including myelin loss and accumulation of perivascular lymphocytes was demonstrated in brain biopsy of levamisole-induced lesions [7].

The toxicological examination of the blood sample from the first inpatient stay showed evidence of the antihelminthic drug levamisole, which is added to cocaine as an adulterant. Since no medical treatment with this substance was carried out at any prior point of time, we assume that this substance was absorbed as part of cocaine consumption. In our opinion, multifocal inflammatory leukoencephalopathy is likely related to the contaminant levamisole rather than a direct toxicity from cocaine as multifocal inflammatory leukoencephalopathy has already been described as an adverse effect of levamisole when being used as antihelminthic drug [1]. In terms of differential diagnosis, we also considered acute disseminated encephalomyelitis (ADEM). However, lack of fever and no evidence of parainfectious or postvaccinal processes do not support ADEM as a differential diagnosis. There is no general consensus on the treatment of toxic leukoencephalopathy. As already described in several case reports, dexamethasone proved to be helpful [1, 8, 11]. Multifocal leukoencephalopathy associated with cocaine use may have an inflammatory basis, possibly related to levamisole contamination, at least in some patients [1]. Wu et al. reported periventricular enhancement, supratentorial lesions and lymphocytic pleocytosis in the cerebrospinal fluid in patients who were treated with levamisole as an immunomodulating adjuvant in chemotherapy with 5-fluorouracil [3]. Though, an additional neurotoxic role of 5-fluorouracil as a cause of the inflammatory reaction must be considered [4].

An early diagnosis of the levamisole-induced multifocal leukoencephalopathy is essential in order to stop exposure and to start treatment with corticosteroids, which is associated with a recovery from the neurological symptoms [3], as seen in our patient.

Vomoos et al. showed that levamisole exposure during the last months in cocaine users is associated with increased executive function impairments. Additionally, a thinning of the lateral prefrontal cortex was suggested. In our patient, a focal or general brain atrophy was not detectable in the follow-up MRI [12].

We assume that cocaine- and levamisole-induced multifocal leukoencephalopathy is underdiagnosed as this disorder is not often described in the literature and anamnesis of drug abuse is not admitted by the patient. Therefore, an additional screening for cocaine and levamisole in clinical practice is useful in similar cases to support the diagnosis.

## Data Availability

Not applicable.

## Footnotes

1 Statement by the State Office of Criminal Investigation in Baden-Wuerttemberg via mail on our request, February 2021.

## References

[1] Vosoughi R, Schmidt BJ (2015). Multifocal leukoencephalopathy in cocaine users: A report of two cases and review of the literature. BMC Neurology.

[2] Solomon N, Hayes J (2017). Levamisole: A high performance cutting agent. Academic Forensic Pathology.

[3] Wu VC, Huang JW, Lien HC, Hsieh ST, Liu HM, Yang CC, Lin YH, Hwang JJ, Wu KD (2006). Levamisole-induced multifocal inflammatory leukoencephalopathy: Clinical characteristics, outcome, and impact of treatment in 31 patients. Medicine (Baltimore).

[4] Hook CC, Kimmel DW, Kvols LK, Scheithauer BW, Forsyth PA, Rubin J, Moertel CG, Rodriguez M (1992). Multifocal inflammatory leukoencephalopathy with 5-fluorouracil and levamisole. Annals of Neurology.

[5] Midthun KM, Nelson LS, Logan BK (2021). Levamisole-a toxic adulterant in illicit drug preparations: A review. Therapeutic Drug Monitoring.

[6] González-Duarte A, Williams R (2013). Cocaine-induced recurrent leukoencephalopathy. The Neuroradiology Journal.

[7] Xu N, Zhou W, Li S, Zhou G, Zhang N, Liang J (2009). Clinical and MRI characteristics of levamisole-induced leukoencephalopathy in 16 patients. Journal of Neuroimaging.

[8] Yan R, Wu Q, Ren J, Cui H, Zhai K, Zhai Z, Duan Q (2013). Clinical features and magnetic resonance image analysis of 15 cases of demyelinating leukoencephalopathy induced by levamisole. Experimental and Therapeutic Medicine.

[9] Blanc, P. D., Chin, C., & Lynch, K. L. (2012). Multifocal inflammatory leukoencephalopathy associated with cocaine abuse: Is levamisole responsible? *Clinical Toxicology (Philadelphia, PA) 50*(6), 534–535; author reply 536. 10.3109/15563650.2012.692794. Erratum in: *Clinical Toxicology (Philadelphia, PA)* 2012, *50*(7), 723. 10.3109/15563650.2012.692794 22702903

[10] Brunt TM, van den Berg J, Pennings E, Venhuis B (2017). Adverse effects of levamisole in cocaine users: A review and risk assessment. Archives of Toxicology.

[11] Lucia P, Pocek M, Passacantando A, Sebastiani ML, De Martins C (1996). Multifocal leucoencephalopathy induced by levamisole. The Lancet.

[12] Vonmoos M, Hirsiger S, Preller KH, Hulka LM, Allemann D, Herdener M, Baumgartner MR, Quednow BB (2018). Cognitive and neuroanatomical impairments associated with chronic exposure to levamisole-contaminated cocaine. Translational Psychiatry.

